# Sex-dependent expression of neutrophil gelatinase-associated lipocalin in aortic stenosis

**DOI:** 10.1186/s13293-022-00480-w

**Published:** 2022-12-12

**Authors:** Eva Jover, Lara Matilla, Ernesto Martín-Núñez, Mattie Garaikoetxea, Adela Navarro, Amaya Fernández-Celis, Alicia Gainza, Vanessa Arrieta, Amaia García-Peña, Virginia Álvarez, Rafael Sádaba, Frederic Jaisser, Natalia López-Andrés

**Affiliations:** 1 Cardiovascular Translational Research, Navarrabiomed (Miguel Servet Foundation), Instituto de Investigación Sanitaria de Navarra (IdiSNA), Hospital Universitario de Navarra (HUN), Universidad Pública de Navarra (UPNA), C/Irunlarrea 3, 31008 Pamplona, Spain; 2 Centre de Recherche des Cordeliers, INSERM, UMRS 1138, Sorbonne Université, USPC, Université Paris Descartes, Université Paris Diderot, Université Paris Cité, 15 rue de l’Ecole de Médecine, 75006 Paris, France; 3 Université de Lorraine, INSERM, Centre d’Investigations Cliniques-Plurithématique 1433, UMR 1116, CHRU de Nancy, French-Clinical Research Infrastructure Network (F-CRIN) INI-CRCT (Cardiovascular and Renal Clinical Trialists), Nancy, France

**Keywords:** Aortic stenosis, Sex, Valve interstitial cells, NGAL, Valvular heart disease, Calcification

## Abstract

**Background:**

Accumulating evidence suggest the existence of sex-related differences in the pathogenesis of aortic stenosis (AS) with inflammation, oxidative stress, fibrosis and calcification being over-represented in men. Neutrophil gelatinase-associated lipocalin (NGAL) is expressed in a myriad of tissues and cell types, and it is associated with acute and chronic pathological processes comprising inflammation, fibrosis or calcification. Sex-dependent signatures have been evidenced for NGAL which expression has been associated predominantly in males to metabolic and cardiovascular disorders. We aimed to analyse sex-related differences of NGAL in AS and its role in the inflammatory and fibrocalcific progression of AS.

**Methods and results:**

220 (60.45% men) patients with severe AS elective for surgical aortic valve (AV) replacement were recruited. Immunohistochemistry revealed higher expression of NGAL in calcific areas of AVs and that was validated by qPCR in in 65 (60% men) donors. Valve interstitial cells (VICs) were a source of NGAL in these samples. Proteome profiler analyses evidenced higher expression of NGAL in men compared to women, and that was further validated by ELISA. NGAL expression in the AV was correlated with inflammation, oxidative stress, and osteogenic markers, as well as calcium score. The expression of NGAL, both intracellular and secreted (sNGAL), was significantly deregulated only in calcifying male-derived VICs. Depletion of intracellular NGAL in calcifying male-derived VICs was associated with pro-inflammatory profiles, dysbalanced matrix remodelling and pro-osteogenic profiles. Conversely, exogenous NGAL mediated inflammatory and dysbalanced matrix remodelling in calcifying VICs, and all that was prevented by the pharmacological blockade of NGAL.

**Conclusions:**

Owing to the over-expression of NGAL, the AV from men may be endowed with higher expression of inflammatory, oxidative stress, matrix remodelling and osteogenic markers supporting the progression of calcific AS phenotypes. The expression of NGAL in the VIC emerges as a potential therapeutic checkpoint, with its effects being potentially reverted by the pharmacological blockade of extracellular NGAL.

**Supplementary Information:**

The online version contains supplementary material available at 10.1186/s13293-022-00480-w.

## Highlights

- NGAL is expressed by inflammatory infiltrates and active VICs in the stenotic AV, being predominantly associated to inflammation, osteogenesis, and calcification.
- The levels of NGAL are higher in VICs and AVs from men than women’s and are associated with pathological features of AS.
- Intracellular NGAL is specifically down-regulated in calcifying male-derived VICs and is associated with inflammation, matrix remodelling and calcification.
- The pharmacological blockade of extracellular NGAL in calcifying male-derived VICs partly prevents from inflammation and matrix remodelling.

**Supplementary Information:**

The online version contains supplementary material available at 10.1186/s13293-022-00480-w.

## Background

Aortic stenosis (AS) is the commonest form of adult valvular heart disease [1]. It is strongly prevalent in the elderly, rising from 2.8% in individuals aged 60–74 to 13.1% in these over their 75s. As per the continuum ageing of the population, valvular heart disease is predicted to become the forthcoming cardiac epidemic [1, 2]. Long pre-clinical fibro-sclerotic stages evolve into calcific AS which precedes the onset of symptoms, and it is a general sign of severity and poor prognosis outcome [3–8]. Aortic valve (AV) replacement or transcatheter valve implantation remain the current therapeutic solutions [9], although are associated to expensive treatments, hospital readmission and reintervention. Altogether, this underscores the unmet clinical need in the management of AS.

The valve interstitial cell (VIC) plays a pivotal role in the pathogenesis of AS by differentiating into aberrant myofibroblast, osteoblast or pericyte-like phenotypes [10, 11]. Substantial histological and basic studies have thoroughly identified inflammation, oxidative stress, apoptosis, angiogenesis, fibrosis and osteogenesis as mechanistic drivers of the pathophysiology of AS [12–14]. Interestingly, important sex disparities seemingly underlie the pathogenesis and phenotypes of AS [15–17]. Indeed, inflammation, oxidative stress, fibrosis and calcification are strongly up-regulated in men, whilst extracellular matrix (ECM) remodelling is rather prevalent in women [18].

Neutrophil gelatinase-associated lipocalin (NGAL, also LCN2) is a pleiotropic glycoprotein that belongs to the lipocalin family and is expressed in a wide range of tissues and cell types, including the AV and the VIC [19–21]. NGAL expression is deregulated in several acute and chronic pathological processes comprising inflammation, fibrosis, cell differentiation and proliferation, iron trafficking or metabolism regulation [19, 20]. NGAL has gained increasing interest as a potent early biomarker for renal injury, inflammation and more recently as a marker of increased cardiovascular risk. Increased levels of NGAL have been reported in atherosclerosis [22], aortic abdominal aneurysm [23] and after MI [24]. Interestingly, the treatment with human recombinant NGAL induces the expression of inflammatory and pro-fibrotic molecules in cardiac and renal fibroblasts [25, 26]. Accordingly, either *Lcn2* gene inactivation or the pharmacological blockade of NGAL have notably proven to ameliorate and prevent the fibrotic and inflammatory responses elicited in humanized in vitro and in vivo models of heart and kidney diseases [25–28]. Moreover, plasma NGAL is associated with serum and aortic root calcification in hemodialized non-diabetic patients, suggesting that NGAL could play a role in cardiovascular calcification [29]. Interestingly, NGAL exerts sex-related functions in different pathological contexts with its expression, predominantly in males, being associated to metabolic and cardiovascular disorders [30–32]. An oxygen-dependent expression of NGAL has been reported in cultured AVs in association with the expression of inflammation and matrix remodelling markers [21]. The role of NGAL has not been evaluated in AS. It is reasonable to speculate that NGAL may enhance the inflammatory, fibrotic, and osteogenic burden predominantly found in men with AS [18].

Here, we aim first to evidence sex-specific differences in the regulation of NGAL in AS and calcifying VICs; and second, to understand the role of NGAL in the context of AS progression towards inflammatory and fibrocalcific phenotypes.

## Materials and methods

### Patient population

Studies were performed using surgical leftover material. The studies were covered by the Research Ethics Committee approval (Pyto.2013/26, num 137) in agreement with the Spanish law (BPCCPMP/ICH/135/95), and the ethical principles recorded in the 1975 Declaration of Helsinki and later amendments. All recruited patients provided informed written consent.

Human AVs were harvested from patients undergoing elective surgical valve replacement at Hospital Universitario de Navarra (Pamplona, Spain). All patients underwent preoperative transthoracic or transoesophageal echocardiography, according to the clinician’s criterion. Peripheral blood samples were collected within the 24 h before the surgery for routine biochemical analyses. Exclusion criteria were: concomitant mitral valve disease, diabetes, endocarditis, chronic kidney disease, malignant tumour or other chronic inflammatory disease.

The harvested AVs were dedicated to histological, immunohistochemical and molecular biology analyses, as well as to VIC isolation for in vitro studies. Moreover, some AVs were macroscopically dissected into healthy, fibrotic or calcified regions for designated experiments, as previously described [33]. In brief, healthy regions defined as macroscopically transparent and pliable tissue not affected by neither thickening nor calcification, were used as control. Fibrotic regions were defined as non-transparent, thickened but pliable tissue. Calcified regions were defined as non-pliable, non-transparent, and visually calcified tissue. All samples were kept at – 80 °C until batch analysis.

### Proteome array profile

Whole AVs from men and women with AS were homogenized in cOmplete™ Lysis-M EDTA-free buffer (Roche, Merck). In brief, 200 µg of total protein/sex were assayed. To that, 25 male-derived AV and 25 female-derived AV were equally pooled (8 µg/donor) and assessed in parallel on dedicated dot-blot membranes (4719964001, Proteome Profiler Human Adipokine Array Kit, ARY024, R&D Systems) by following the manufacturer’s instructions. Dot blot densitometric analyses were performed using Image Lab software. The negative spots were used for background subtraction to all the dot blots. Dot blots corresponding to duplicates of specific targets were then normalized to the averaged densitometry of 3 pairs of reference spots as per the manufacturer’s instructions.

### Cell isolation and culture

Human VICs were isolated and expanded from AVs harvested during elective surgical valve replacement interventions (6 biological replicates/sex), as previously published [34]. In brief, AV were minced and incubated in a Collagenase type 2 (240 U/mg) buffered-solution (Worthington Biochemical Product, country) for 1 h, 37 °C in a saturation humidified 5% CO_2_ incubator. VICs were pelleted at 350 g, 5 min and expanded onto 2% gelatin-coated flasks using DMEM/F12 media (Lonza,) supplemented with 20% FBS (Gibco), 100 U/mL Penicillin (Lonza), 100 ug/mL streptomycin (Lonza), 10 ng/mL FGF-2 (Novus Biologicals) and 5 µg/mL insulin (Sigma-Aldrich). All experiments were conducted in technical triplicates unless otherwise indicated.

### In vitro osteogenesis

Procalcifying media was prepared in DMEM 1 g/L glucose media supplemented with 1% FBS and 2.6 mM Na_2_HPO_4_/NaH_2_PO_4_, pH 7.4 buffer (high inorganic phosphate buffer, namely HP). VICs were used at passages 3–5 for all experiments and media were replaced every 2 days. Moreover, VICs were challenged with 500 ng/mL recombinant human NGAL (rhNGAL) (R&D systems) or 100 nM GP-1 (GPZ614741) compound (chemical inhibitor for NGAL signalling) [26].

### Silencing of NGAL

A pool of 4 small interfering RNA (siRNA) against *LNC2*/NGAL (siNGAL) were transiently transfected in control and calcifying VICs using a magnet assisted transfection (MATra) system (IBA BioTAGnology). Scramble sequences were used as negative control. Both scramble and siNGAL were used at 30 nM in OPTIMEM (Gibco) following the manufacturer’s instructions.

### Calcification assessment

VIC calcification was assessed using the *o*-cresolphthalein method (MAK022, Merck Sigma). Calcium deposits were collected as 0.6 N HCl extracts, as previously published [35, 36]. Cell monolayers were homogenized in 0.1% SDS, 0.1 M NaOH, 5 mM EDTA buffer for ulterior normalization of the calcification readouts. VICs were assayed in technical quadruplicates.

### Enzyme-linked immunosorbent assays (ELISA)

The following factors were analysed in AV lysates and cell supernatants: NGAL, interleukin (IL)-1β, IL-6, IL-8, CCL-2/MCP-1, CCL-5/RANTES, CD14, CD44, intercellular adhesion molecule 1 (ICAM-1), galectin-3, myeloperoxidase, tissue inhibitor of metalloproteinases (TIMP)-1, TIMP-2, metalloproteinase (MMP)-2, MMP-9, collagen type I (COL1), bone morphogenetic protein (BMP)2, BMP4, BMP9, periostin, osteopontin. All purchased from R&D Systems. Cell supernatants were collected and centrifuged at 10,000*g* for 3 min, at 4 °C to remove cell debris and were kept at − 80 °C until batch analysis. Immunoreactive levels were normalized to total protein concentration.

### Protein isolation and western blotting

Whole cell protein lysates were prepared in RIPA buffer (Santa Cruz), following the manufacturer’s protocols. Total protein (8 µg) was resolved onto native or denaturing 4–15% TGX Stain-Free™ precast SDS-PAGE, as appropriate, and blotted onto 0.2 µm pore size Hybond-c Extra nitrocellulose membranes (Bio-Rad). Primary antibodies were incubated overnight at 4 °C. Stain-free imaging or β-actin immunoblotting were used as a loading controls. Malondialdehyde and NGAL (both from abcam) were used as primary antibodies; Amersham™ ECL™ Anti-rabbit and anti-mouse IgG HRP were used as secondary antibodies. Densitometric band analysis was performed using Image Lab software (Bio-Rad). All antibodies and working concentrations are listed in Additional file 1: Table S1.

### RNA isolation, RT and qPCR

Total RNA was isolated according to a standardized phenol–chloroform protocol, using Qiazol reagent and miRNeasy mini Kit (217004, QIAGEN, Germany), and reverse-transcribed into single-stranded cDNA, using an iScript Advanced cDNA Synthesis Kit (Bio-Rad). Downstream qPCR amplification was performed using iQ SYBR Green Supermix (Bio-Rad) in an CFX Connect Real-Time PCR System (Bio-Rad). The relative expression of each selected gene product was calculated using the 2^−ΔΔ*Ct*^ method. All reactions were performed in technical triplicates. All primers are listed in Additional file 1: Table S2.

### Histological and immunohistochemistry

Harvested AVs were fixed in 10% neutral buffered formalin, and embedded in paraffin after gross decalcification in 10% formic acid solution for 24 h. Five-μm sections were deparaffinized to distilled water. Movat pentachrome staining was performed for a general histological characterization (Abcam), following the manufacturer’s instructions. Immunohistochemistry techniques were applied using an automated immunostainer Leica BOND-Polymer Refine Detection (Leica), following the manufacturer’s protocols. The primary antibodies used and working concentrations are listed in Additional file 1: Table S1. Secondary antibodies poly-HRP-anti-mouse or poly-HRP-anti-rabbit IgG were used, as appropriate. Positive immunoreactive signal was developed using an enhanced 3,3′-diaminobenzidine system (Leica). Slides were mounted using DPX mounting media (Merck/Sigma-Aldrich).

### Statistical analysis

Normal distribution was assessed by Kolmogorov–Smirnov. Continuous variables are shown as mean ± standard error of the mean (SEM) or median (IQR) depending on their distribution. Categorical variables are presented as percentages. Normally distributed variables were analysed using the univariate two-tailed Student’s *t* test (two group comparison) or one-way analysis of variance (multiple comparisons ANOVA), as appropriate. ANOVA post hoc analysis included Tuckey or T3 Dunnett testing, as appropriate. Non-parametric tests, including the Wilcoxon/Mann–Whitney *U* test or the Kruskal–Wallis test, were used for data not normally distributed. Statistical significance was accepted at *p* < 0.05. Categorical variables were expressed as percentages and compared using χ^2^-test, or Fisher exact test, as appropriate. Analyses and graph plotting were performed using GraphPad Prism 6.0 or SPSS 19.0 for Windows statistical packages.

## Results

### NGAL expression in calcific AV stenosis

The baseline clinical and demographic characteristics of AS patients recruited for this study are shown in Table 1. Whole AVs (*n* = 220, 60.45% men) were used to study sex-related differences in the expression of NGAL and its association with underlying pathogenic mechanisms. In 65 donors (60% men), additional analyses were performed to study how the expression of NGAL transcript was associated with fibrosis or calcification. As expected, women were significantly older than men (75.5 [68–80] vs 71 [65–77] years, *p* = 0.0008), in agreement with their late clinical onset of AS symptoms. Moreover, lower height, weight and body surface were reported in women (all of them *p* < 0.0001). The use of diuretics was significantly higher in women (63.10% vs 49.22%, *p* = 0.0499), whilst statin intake was higher in men (73.44% vs 48.81, *p* = 0.0004).

**Table 1:** ^Clinical and demographic data of the AS cohort^

Variables	Total	Men	Women	*p *value
*n* (%)	220 (100)	133 (60.45)	87 (39.54)	
Age (median [IQR])	72 [66–78]	71 [65–77]	75.5 [68–80]	0.0008
Height (median [IQR])	163 [156–170]	168 [163–172]	154 [150–157.8]	< 0.0001
Weight (mean ± SD)	77.11 ±14.30	82.18 ± 12.49	69.40 ± 13.47	< 0.0001
Body surface (mean ± SD)	1.81 ± 0.19	1.91 ± 0.15	1.67 ± 0.16	< 0.0001
DM, *n* (%)	63 (29.72)	40 (31.25)	23 (27.38)	0.6452
HTA, *n* (%)	149 (70.28)	91 (71.09)	58 (69.05)	0.7607
Renal insufficiency, *n* (%)	14 (6.60)	8 (6.25)	6 (7.14)	0.7852
NYHA class, *n* (%)				
I	22 (10.78)	17 (13.49)	5 (6.41)	
II	123 (60.29)	83 (65.87)	40 (51.28)	
III	55 (26.96)	25 (19.84)	30 (38.46)	
IV	4 (1.96)	1 (0.79)	3 (3.85)	
Drug medicines				
ACEI, *n* (%)	53 (25.24)	32 (25.20)	21 (25.30)	1.000
ARB, *n* (%)	55 (26.19)	35 (27.60)	20 (24.10)	0.6320
Diuretics, *n* (%)	116 (54.72)	63 (49.22)	53 (63.10)	0.0499
B-blockers, *n* (%)	59 (27.83)	36 (28.13)	23 (27.38)	1.000
Statins, *n* (%)	135 (63.68)	94 (73.44)	41 (48.81)	0.0004
Biochemical analyses				
BNP, pg/mL (median [IQR])	128 [56.5–250]	109.5 [49.5–231.0]	144 [67–263.5]	0.1671
Triglycerides, mg/dL (median [IQR])	97 [76–131]	95.5 [71.5–130.8]	104 [83.5–131]	0.1652
HDL, mg/dL (median [IQR])	45 [37–57]	43 [35–53.75]	49 [38.5–61.0]	0.0372
LDL, mg/dL (mean ± SD)	107.1 ± 34.15	100.1 ± 32.76	118.0 ± 33.58	0.0003
Echocardiographic parameters				
BAV, *n* (%)	69 (48.25)	39 (49.37)	30 (46.88)	0.8666
Max gradient (median [IQR])	74 [65.3–88.5]	73 [66–88]	77 [65–89]	0.6082
Mean gradient (median [IQR])	47 [41–57]	47 [41–56]	48 [41–58.5]	0.5000
Valvular area echocardiography, cm^2^ (median [IQR])	0.72 [0.6–0.9]	0.8 [0.7–0.9]	0.7 [0.6–0.8]	0.0024
End-systolic volume (cc) (median [IQR])	41 [25–58.5]	45.00 [30.25–63.25]	33 [18.5–55.0]	0.0039
EF % (median [IQR])	67 [58–74]	66 [57–73]	67 [59–76.5]	0.3813
Calcium score (A.U.) (median [IQR])	2523 [1785–5116]	3128 [2118–5367]	2090 [1504–3888]	0.0471

*N *sample size (biological replicates), *SD* standard deviation, *IQR* interquartile range, *DM* diabetes mellitus, *HTA* arterial hypertension, *NYHA class* New York Heart Association Classification for Heart Failure, *ACEI* angiotensin converting enzyme inhibitors, *ARB* angiotensin II receptor blockers, *BNP* brain natriuretic peptide, *HDL* high density lipoproteins, *LDL* low density lipoprotein, *BAV* bicuspid aortic valve, *EF* ejection fraction, *A.U.* Agatston units

**Fig. 1:**
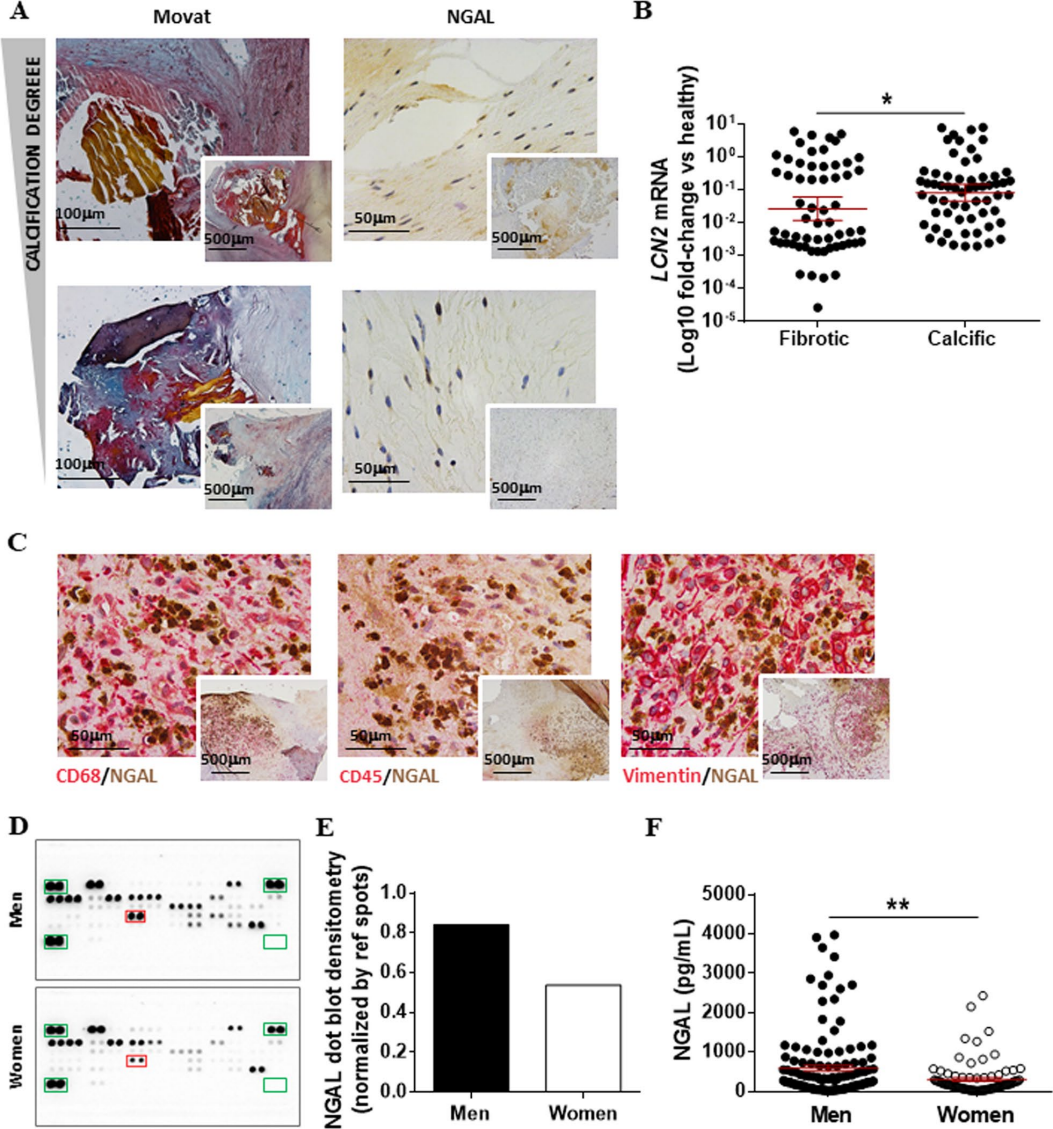
Sex-related expression of NGAL in AVs harvested from AS patients. **A** Representative microphotographs of AVs harvested from patients undergoing elective surgical valve replacement with different degrees of calcification. AVs were characterized by Movat staining and NGAL immunohistochemistry. **B** Transcript expression of *LCN2* mRNA (NGAL gene) in AVs (*n* = 65) sectioned into fibrotic and calcific areas. *18S*, *GAPDH*, *ACTA2* and *HPRT* were used as housekeeping genes and data are plotted as log10-fold changes versus the expression in healthy sections. **C** Representative microphotographs of AVs from AS patients showing co-localization of NGAL with macrophages (CD68), leucocytes (CD45) and VICs (vimentin). **D** Proteome profiler array dot blots from men and female-derived stenotic AVs (*n* = 25 donors pooled per sex). NGAL blots are red-squared within the dot-blot membranes. Reference spots and negative spots are green-squared within the dot-blot membranes. **E** Densitometric analyses were plotted upon normalization by the reference spots (internal controls) included within each dot-blot membrane. **F** Scatter dot plot for the expression of NGAL (pg/mL) in whole AVs harvested from men (*n* = 133) and women (*n* = 87). Mean ± SEM are shown in red colour within the scatter blots. *p < 0.05; **p < 0.01. *NGAL* Neutrophil Gelatinase-Associated Lipocalin, *LCN2* NGAL codifying gene, *CD* cluster of differentiation

NGAL has been broadly associated with cardiac fibrosis and remodelling [25, 37] and might contribute to the progression of calcific AS. AVs with different degrees of calcification were assessed for the expression of NGAL. Representative pictures of high and mild calcified AVs have been displayed in parallel with the corresponding NGAL immunohistochemistry (Fig. 1A). The expression of NGAL was higher in more calcified samples. We next studied the expression of NGAL at the transcript level in AV (*n* = 65, 60% men) dissected into fibrotic and calcific areas. NGAL expression was significantly higher in calcific sections than in the fibrotic counterparts (0.75 ± 0.2 vs. 0.61 ± 0.2, *p* = 0.0409) (Fig. 1B). Double immunohistochemical analyses were performed to reveal whether the expression of NGAL is exogenously produced by recruited inflammatory infiltrates or by the VIC. Our results showed that inflammatory infiltrates, mostly as macrophages (DC68) and leukocytes (CD45), were an important but not the solely source of valvular NGAL expression (Fig. 1C). Concomitant expression of vimentin and NGAL proved that activated VICs, pivotal to the progression of AS, were an additional source of NGAL in the stenotic AV.

### Sex-related expression of NGAL in aortic stenosis

Accumulating evidence suggest sex-specific differences in the pathogenesis of AV stenosis with enhanced inflammation, fibrosis, apoptosis and osteogenic cues in males compared to females [18]. Therefore, we next studied whether NGAL expression may be sex-related in AS. Whole AVs from 25 men and 25 women were randomly selected and pooled to be assayed using a proteome profiler array (Fig. 1D). NGAL expression was 1.6 times up-regulated in men compared with women. These results were further validated by ELISA in 131 men and 79 women (592 ± 74 vs. 306 ± 45, *p* = 0.0068) (Fig. 1E, F).

### NGAL is correlated with the expression of inflammation, oxidative stress, extracellular matrix remodelling and osteogenesis markers in the stenotic AV

**Fig. 2:**
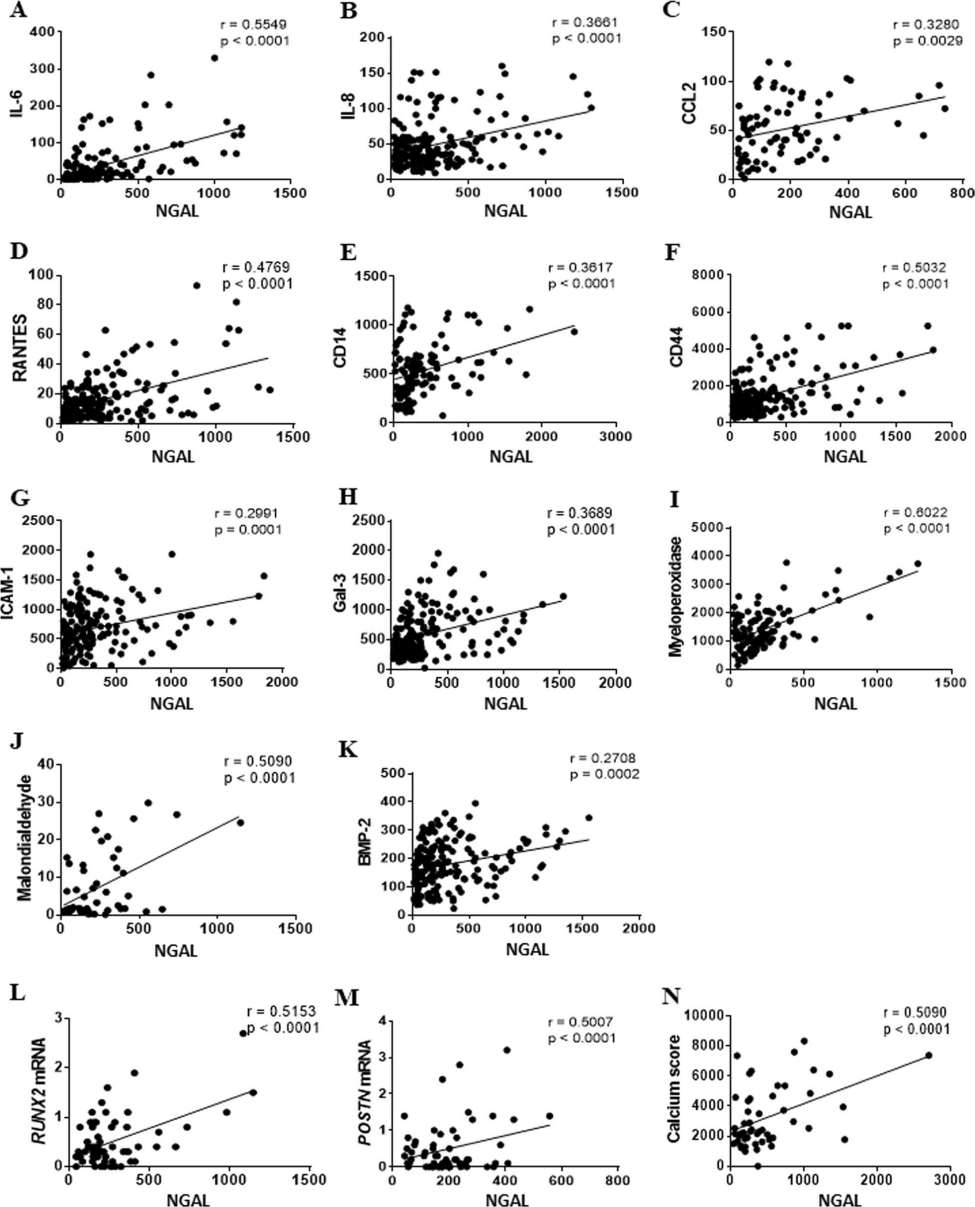
Expression of NGAL in AVs is associated with inflammation, oxidative stress, and osteogenesis. Correlation dispersion plots for NGAL expression with inflammatory markers IL6 (**A**), IL8 (**B**), monocyte chemoattractant protein-1 CCL2 (**C**), RANTES (**D**), CD14 (**E**), CD44 (**F**), ICAM-1 (**G**) and Gal-3 (**H**); oxidative stress markers myeloperoxidase (**I**) and malondialdehyde (**J**); and osteogenic markers BMP2 (**K**), *RUNX2* mRNA (**L**), Periostin mRNA (**M**) and calcium score evaluated by CT scan prior to the surgical valve replacement (**N**). Correlations were performed in stenotic AVs (*n* = 220). *X*-axis labels are referred to NGAL (pg/mL) measured in AV protein homogenates. IL, interleukin; CCL2, chemokine (C–C motif) ligand 2 (CCL2) or monocyte chemoattractant protein 1 (MCP1). *CD* cluster of differentiation, *ICAM-1*, Intercellular Adhesion Molecule 1, *Gal-3*, galectin-3, *BMP* bone morphogenetic protein, *RUNX2* Runt-related transcription factor 2, *POSTN* periostin

Since NGAL was found associated to inflammatory cells and calcific areas, we further studied the correlation of NGAL with specific markers of pathogenic mechanisms in stenotic AVs. Importantly, NGAL was positively correlated with the inflammatory markers IL-6 (Fig. 2A), IL-8 (Fig. 2B), CCL-2/MCP1 (Fig. 2C), RANTES (Fig. 2D), CD14 (Fig. 2E), CD44 (Fig. 2F), ICAM-1 (Fig. 2G) and Galectin-3 (Fig. 2H). In line with these results, NGAL was also correlated with enhanced oxidative stress markers myeloperoxidase (Fig. 2I) and malondialdehyde (Fig. 2J).

Importantly, NGAL expression was significantly associated with the expression of BMP2 (Fig. 2K), *RUNX2* mRNA (Fig. 2L), *POSTN* (periostin) mRNA (Fig. 2M) and the calcium score (Agatston score) (Fig. 2N).

### Deregulated expression of intracellular and extracellular NGAL in calcifying VICs

**Fig. 3:**
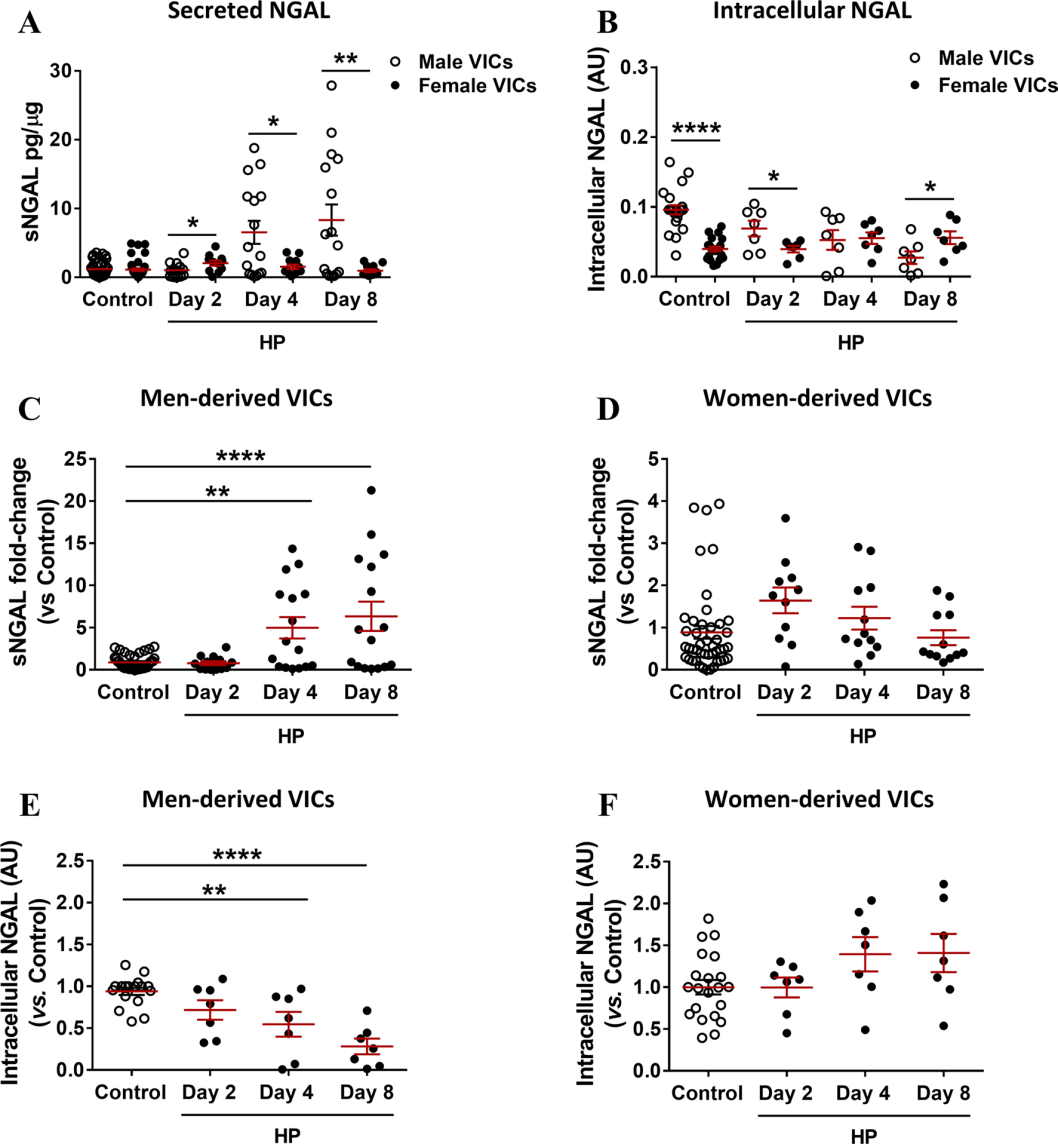
Expression of NGAL in calcifying VICs isolated from men and women donors undergoing AV replacement. Data are presented as scatter dot plots, mean ± SEM (shown in red colour). Cultured VICs were HP-challenged for 2, 4 and 8 days. Supernatants and protein cell lysates were harvested at the stated time points. Side-by-side sex comparison of the secreted NGAL (sNGAL) measured by ELISA in cell supernatants from calcifying male (*n* = 6) and female-derived VICs (*n* = 6). sNGAL was normalized to the total yield of protein cell monolayer (pg/µg) (**A**). Side-by-side sex comparison of the intracellular NGAL in arbitrary units (AU) upon normalization to the stain free (**B**). Fold-change analyses of the sNGAL expression in male-derived VICs (*n* = 6) (**C**) or female-derived VICs (*n* = 6) (**D**). Fold-change analyses of the intracellular NGAL in calcifying male-derived VICs (*n*=6) (**E**) or female-derived VICs (*n* = 6) (**F**). Band densitometries were normalized to stain free and are plotted as a fold-change relative to Control condition. Control condition within each sex was used as calibrator condition for statistical comparisons in **C**−**F**. *AU* arbitrary units, *HP* pro-calcifying high phosphate media conditioning (2.6 mM Pi), *Ctrl* control, *NGAL* Neutrophil Gelatinase-Associated Lipocalin. *p < 0.05; **p < 0.01; ****p < 0.0001

We developed an in vitro calcification model on primary cultured VICs (Additional file 2: Fig. S1A). The expression of NGAL was studied at the intracellular and extracellular levels (Fig. 3). In brief, VICs from men and women with AS were HP-challenged for 2, 4 and 8 days. At the stablished time points, supernatants and protein cell lysates were harvested to study the effect of HP-challenge on the expression of NGAL in male or female-derived VICs at the extracellular and intracellular levels, respectively. Side-by-side comparisons of the net values of NGAL expression (pg/ug or A.U., as appropriate) were analysed in Fig. 3A, B. Statistical differences were reported by comparing the expression of secreted NGAL (Fig. 3A) or intracellular NGAL (Fig. 3B) at each time point between sexes. Moreover, statistical comparisons within each sex were performed. Further analyses of the fold-changes of NGAL expression sex were separately analysed within each in Fig. 3C–F using their respective control conditions as calibrator for the statistical comparisons.

The levels of secreted NGAL (sNGAL) in male or female-derived VICs under control conditions were not statistically different (male: 1.19 ± 0.19 vs female: 1.11 ± 0.29 pg/µg, *p* = 0.9756). At day 2, the content of sNGAL in the secretome of female-derived VICs was higher than in male-derived VICs (male: 1.03 ± 0.3 vs female: 2.06 ± 0.4 pg/µg, *p* = 0.0401), whilst at day 4 and 8 the secretion of sNGAL was consistently higher in male-derived VICs than in female-derived VICs (day 4, male: 6.54 ± 1. 7 vs female: 1.54 ± 0.3 pg/µg, *p* = 0.0170; day 8, male: 8.32 ± 2.3 vs female: 0.95 ± 0.2 pg/µg, *p* = 0.0097) (Fig. 3A). The level of sNGAL was only up-regulated in calcifying male-derived VICs (ANOVA *p* < 0.0001) (Fig. 3C), with no significant differences seen in calcifying female-derived VICs (ANOVA *p* = 0.0796) (Fig. 3D). sNGAL was significantly up-regulated at days 4 (net values: 1.19 ± 0.19 vs 6.54 ± 1.67 pg NGAL/µg protein, *p* = 0.0018) and 8 (net values: 1.05 ± 0.37 vs 8.32 ± 2.27 pg NGAL/µg protein, *p* < 0.0001) (Fig. 3A, C). Additional, linear trend analyses revealed a significant and progressive increase of sNGAL levels only in male-derived VICs (*p* < 0.0001), not seen in female-derived ones (*p* = 0.4334).

Interestingly, the intracellular expression of NGAL was 2.46 times higher in control male VICs than female’s (0.096 ± 0.007 vs 0.039 ± 0.003 AU, *p* < 0.0001) (Fig. 3B). Nevertheless, the expression of intracellular NGAL in HP-challenged VICs from women, less calcific than men’s (data not shown), was 2.07 times higher than in male VICs at day 8 (0.027 ± 0.009 vs 0.056 ± 0.009, *p* = 0.0444) (Fig. 3B). Intracellular NGAL was progressively down-regulated in HP-challenged VICs from men (Fig. 3E). The expression of intracellular NGAL in female-derived VICs did not show significant modifications at specific time points (Fig. 3F). Additional linear trend analyses within each sex confirmed that intracellular NGAL progressively decreased over time (*p* < 0.0001) in calcifying male-derived VICs (*n* = 6), while it increased over time in female-derived VICs (*n* = 6) (*p* = 0.0148).

Our results evidenced a significant regulation of NGAL expression only in male-derived VICs. Accordingly, the experiments dedicated to study the role of exogenous NGAL, its pharmacological inhibition and its intracellular inhibition were exclusively performed in male-derived VICs.

### Depletion of intracellular NGAL is associated with pro-inflammatory phenotypes, dysbalanced matrix remodelling and pro-osteogenic phenotypes in the VIC

**Fig. 4:**
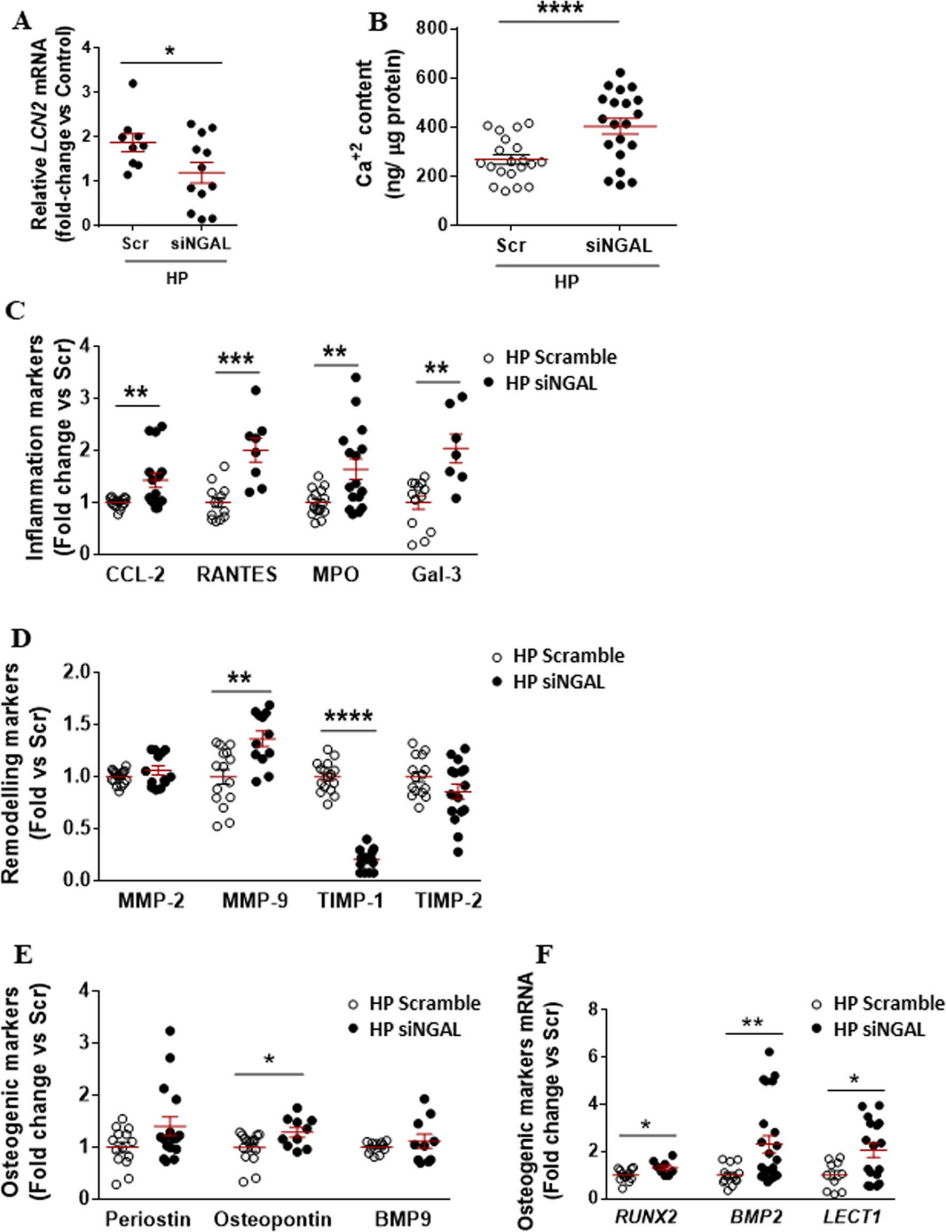
Effect of NGAL silencing on calcifying VICs from men donors undergoing elective surgical AV replacement. NGAL silencing was evidenced at transcript levels in calcifying male-derived VICs. Scramble (Scr) control group was used as calibrator for the fold-change calculations. Scatter dot plots for the relative expression of NGAL codifying gene (*LCN2*) in calcifying male-derived VICs (**A**). *GAPDH*, *18S*, *HPRT* and *ACTA2* were used as housekeeping genes. Scatter dot plot of calcium deposits in calcifying Scr and siNGAL male-derived VICs (**B**). Markers of inflammation CCL-2, RANTES, myeloperoxidase and galectin-3 (**C**); ECM remodelling markers MMP-2, MMP-9, TIMP-1 and TIMP-2 (**D**); and osteogenesis (**E**) were assessed by ELISA in cell supernatants from siNGAL VICs under HP conditions. Osteogenic markers *RUNX2* and *BMP2* were analysed at the mRNA transcript level as well as the quiescent VIC marker *LECT1* for chondromodulin (Chm)-I (**F**). *AU* arbitrary units; *p < 0.05; **p < 0.01; ***p < 0.001; *p* < 0.0001. *Scr* scramble, *siNGAL* small interfering RNA against NGAL, *LCN2* NGAL codifying gene, *CCL2* chemokine (C–C motif) ligand 2 (CCL2) or monocyte chemoattractant protein 1 (MCP1), *MPO* myeloperoxidase, *Gal-3* galectin-3, *MMP* metalloproteinase, *TIMP* tissue inhibitor of metalloproteinases, *BMP* bone morphogenetic protein, *LECT1* chondromodulin (Chm)-I codifying gene; periostin; *RUNX2* Runt-related transcription factor 2

In order to mimic the specific down-regulation of intracellular NGAL reported in calcifying male-derived VICs, siRNA approaches were used. NGAL silencing was partly achieved as demonstrated at transcript level in control (Additional file 2: Fig. S2A) and calcifying VICs (Fig. 4A), as well as at the protein level in control conditions (Additional file 2: Fig. S2B). Silencing of NGAL was associated with an increased calcification (Fig. 4B) compared with scramble-transfected VICs. The expression of inflammatory cues was consistently up-regulated in calcifying NGAL-silenced VICs as evidenced by enhanced secretion of CCL-2, Rantes, myeloperoxidase and Galectin-3 (Fig. 4C). These findings were also found in control NGAL-silenced VICs (Additional file 2: Fig. S2C). ECM remodelling was also dysregulated in calcifying NGAL-silenced VICs, as shown by a significant up-regulation of MMP-9 paralleled by the down-regulation of TIMP-1 (Fig. 4D). Importantly, silencing of NGAL in control VICs was associated with an enhanced expression of MMP-9 and MMP-2, and a diminished expression of TIMP-1 and TIMP-2 (Additional file 2: Fig. S4D). Moreover, the levels of osteopontin were higher in calcifying NGAL-silenced VICs than scramble ones (Fig. 4E). Moreover, the expression of *RUNX2*, *BMP2* and *LECT1* (chondromodulin-I gene) transcripts was higher in calcifying NGAL-silenced VICs than in calcifying scramble VICs (Fig. 4F). Similar effects were seen in NGAL-silenced VICs under control conditions for the expression of osteopontin and *BMP2* mRNA (Additional file 2: Fig. S2E, F, respectively).

### Effect of the exogenous supplementation of NGAL and the pharmacological blockade of NGAL on the expression of inflammation, ECM remodelling and osteogenesis markers in calcifying male-derived VICs

To unravel whether extracellular NGAL exerts additional activation of calcifying male-derived VICs, these were challenged with rhNGAL for 4 days. Countless publications have evidenced that NGAL stands as a potential therapeutic target in different pathological sets, including cardiovascular disease. Recently, GP-1 compound was proven to diminish the fibro-inflammatory effects elicited by NGAL in in vitro and in vivo models of kidney and heart disease [26]. Accordingly, calcifying VICs were treated with GP-1 to analyse the effect pharmacological blockade of NGAL on the expression of inflammation, remodelling and calcification markers.

**Fig. 5:**
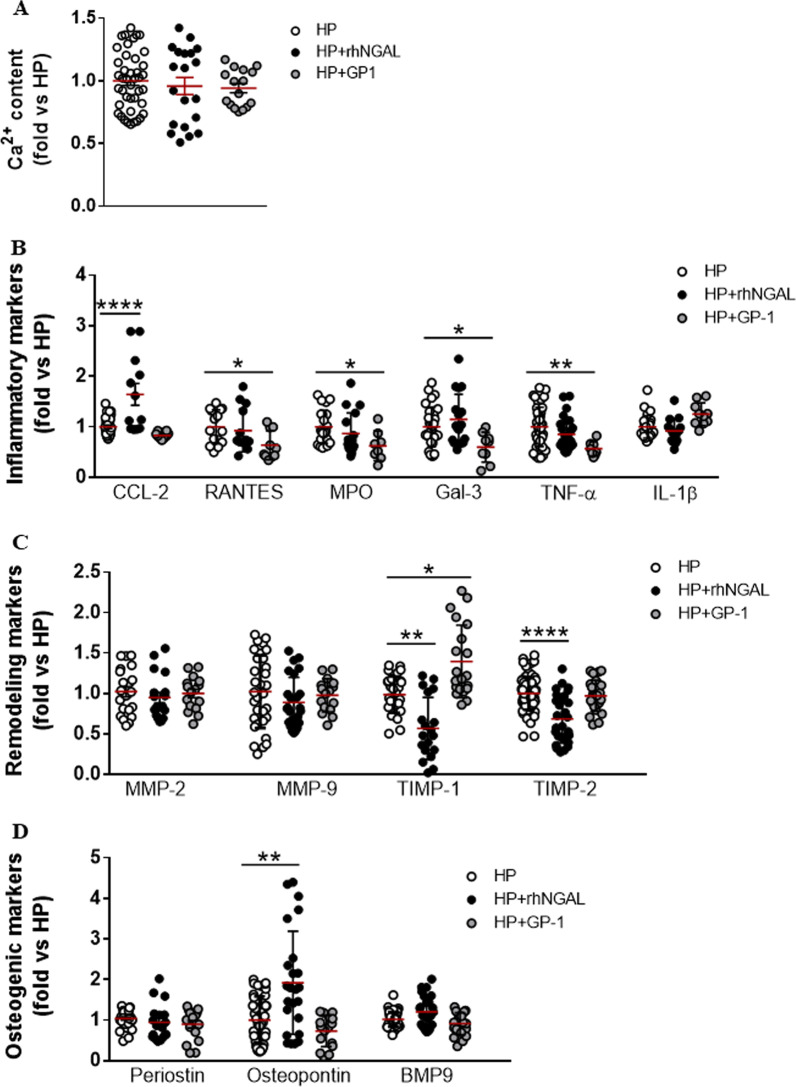
Effect of exogenous NGAL regulation in calcifying VICs isolated from men donors. Scatter dot plot of calcium deposits in calcifying VICs isolated from men and challenged with HP, HP + 500 ng/mL rhNGAL and HP + 100 µM GP-1 (**A**). Markers of inflammation (**B**), ECM remodelling (**C**) and osteogenesis (**D**) were assessed by ELISA in cell supernatants from siNGAL VICs under HP conditions. All comparisons have been represented as a fold change relative to Control condition. *p < 0.05; **p < 0.01; ****p < 0.0001. CCL2, chemokine (C–C motif) ligand 2 (CCL2) or monocyte chemoattractant protein 1 (MCP1). *MPO* myeloperoxidase, *Gal-3* galectin-3, *TNF-a* tumour necrosis factor-a, *IL* interleukin, *MMP* metalloproteinase, *TIMP* tissue inhibitor of metalloproteinases, *BMP* bone morphogenetic protein

RhNGAL supplementation or GP-1 treatment did not have any statistical effect on the calcification of male-derived VICs challenged with HP (Fig. 5A). Importantly, rhNGAL increased CCL-2 levels without exerting major changes in other pro-inflammatory mediators in calcifying VICs. Nevertheless, GP-1 decreased the pro-inflammatory response elicited in calcifying VICs. The levels of Rantes, myeloperoxidase, galectin-3 and TNF-α were significantly lower in calcifying VICs treated with GP-1(Fig. 5B). Moreover, whereas rhNGAL consistently down-regulated the expression of TIMP-1 and TIMP-2, their expression was recovered when supplementing GP-1 to calcifying VICs, although it only reached the statistical significance for TIMP-1 (Fig. 5C). Exogenous supplementation of rhNGAL was associated with a significant up-regulation of osteopontin (Fig. 5D). GP-1 modestly down-regulated osteopontin and BMP9 (Fig. 5D), but, in agreement with our calcification results (Fig. 5A), it did not reach the statistical significance.

All these results were not reported in control rhNGAL-challenged VICs (Additional file 2: Fig. S3A, B), except for osteopontin (Additional file 2: Fig. S3C).

## Discussion

NGAL plays a major role in inflammatory and fibrotic processes, and increased levels of NGAL correlate with symptomatic cardiovascular diseases, atherosclerosis risk factors, disease severity burden and mortality [38–40]. Our results show that NGAL may also play a role in AS. This is the first study evidencing the expression of NGAL in calcific AS and calcifying human VICs; the main findings being (1) NGAL expression was higher in men than women and was associated with enhanced inflammatory, oxidative stress, osteogenic and calcific burden in AS; (2) VICs were a source of NGAL significantly modulated only in in vitro calcifying male-derived VICs; (3) the subcellular location of NGAL, extracellular or intracellular, was associated with the activation of different pathological cues that (4) can be partly mitigated by the pharmacological blockade of extracellular NGAL. Altogether, our results suggest a sex-related role of NGAL in the progression of calcific AS and propose NGAL as a druggable target to prevent the VIC-to-osteoblast differentiation predominantly found in men.

NGAL is broadly expressed and participates in pathological inflammation, fibrosis, cell differentiation, immune responses or metabolism [19, 20, 41]. Initially identified in neutrophils [42], NGAL is also expressed by epithelial cells, renal tubular cells, hepatocytes, cardiomyocytes, endothelial cells, vascular smooth muscle cells, fibroblasts, macrophages or dendritic cells [37, 40]. We identified that leukocytes and macrophages were an important but not the solely source of valvular NGAL. Activated VICs additionally expressed NGAL within the stenotic AV. Grande-Allen et al. [21] preliminarily described the expression of NGAL in hypoxia-challenged VICs in porcine and human specimens. Herein, we extend these findings by demonstrating that NGAL was associated with calcification, even within the same AV, and that the expression of NGAL was higher in men than women. Besides, we evidenced an important sex-dependent signature on the expression of NGAL that was significantly modulated in calcifying male-derived VICs, but not in calcifying female-derived VICs. Importantly, side-by-side sex comparisons evidenced no significant differences on the basal expression of sNGAL between male and female-derived VICs. These results further reinforce the idea that either the male-derived VICs is more sensitive to pro-calcifying environments, or the female-derived VIC is more resistant to it favouring sex-specific phenotypes in AS. Conversely, the expression of intracellular NGAL was significantly higher in male than female-derived VICs in basal conditions, being strongly down-regulated by the HP-challenge only in male VICs. Considering the deleterious effect elicited by the cellular depletion of NGAL in HP-challenged VICs, the progressive up-regulation of intracellular NGAL in HP-challenged female-VICs is provoking. Future studies will be necessary to understand whether the up-regulation of intracellular NGAL in female VICs may stand for a weakened response to pro-calcific challenges in comparison to male-derived VICs.

Moreover, the subcellular location of NGAL, intracellular or extracellular, endowed the male-derived VIC with different pathologic phenotypes supporting inflammation, ECM remodelling and further osteogenic differentiation. The impact of these results is twofold. First, it clearly reinforces the singularity of AS as independent pathological entity compared to calcific forms of atherosclerosis. Second, inhibiting the expression of intracellular NGAL may not be a therapeutic solution in AS. Previous publications have evidenced that the effects of intracellular and extracellular NGAL may or may not overlap [43, 44]. Tong et al. [44] showed that only NGAL silencing was able to increase the cell death of A549 lung cancer cells, with no effects whatsoever reported for rhNGAL. Moreover, although the effect of NGAL pharmacological blockade using GP-1 on renal fibrosis mimicked the effect of global genetic inactivation of *Lcn2*, it did not improve either renal inflammation or function [26]. Silencing of NGAL and subsequent down-regulation of NGAL was associated with a further worsened phenotype to that triggered by pro-calcific challenge in men VICs. While NGAL depletion seems to be overall protective for the cardiovascular and renal systems, it does not prevent acute kidney injury [26].

Our ex vivo analyses on human AVs showed a higher expression of NGAL in men than women AVs. Such sex-dependent differences, although scarce, have been previously demonstrated in different pathological contexts, with worsened outcomes predominantly in males. In a coronary artery ligation model of myocardial infarction, circulating levels of NGAL were higher in male rats than in female rats paralleling the enhancement of inflammation [32]. Moreover, only in males, plasma NGAL was correlated to the infarct size and signs of heart failure [45], thus supporting the sex-specific role of NGAL in the cardiovascular context. Plasma NGAL was preliminarily shown to exhibit a sex-specific profile in healthy subjects and was associated with cardiometabolic risk factors [46]. Furthermore, higher plasma NGAL is associated with greater metabolic derangements, blood pressure and triglycerides levels in male children born upon in vitro fertilization [31]. Accordingly, our data revealed that higher NGAL expression in stenotic AVs and VICs from men paralleled the expression of inflammation, fibrosis and calcification markers. Conversely, NGAL expression seems to be sex-specific in mice adipose tissue by promoting inflammation and fibrosis only in female mice [30, 47].

Inflammation is acknowledged an early mechanism triggering AS [12, 48] with a major role in the calcification of male-derived VICs [49, 50] and in the development of dominant calcific phenotypes in men with AS. NGAL expression was strongly associated with the inflammatory and oxidative stress markers Il-6, Il-8, CCL2, Rantes, CD14, CD44, ICAM-1, galectin-3, myeloperoxidase or malondialdehyde. In recent publications, we thoroughly demonstrated that these markers are significantly up-regulated in men with AS compared to women [18, 51]. Both inflammatory and pro-fibrotic phenotypes are induced in human cardiac fibroblasts by rhNGAL and are abolished by the pharmacological blockade of NGAL with GP-1 [25]. In vitro, rhNGAL only enhanced the expression of CCL2 in calcifying male-derived VICs. Nevertheless, the up-regulation of inflammatory markers such as Rantes, myeloperoxidase, TNF-α, IL1β and galectin-3 was eluded in calcifying VICs upon GP-1 treatment. Secreted NGAL may act as an inflammatory cytokine in AS, likely contributing to the creation of a cascade of events that result in a dysfunctional microenvironment [52, 53]. For example, NGAL may elicit paracrine and chemoattractant effects on circulating inflammatory cells and other cardiovascular cells [20], thus perpetuating the continuum inflammatory cell recruitment, fibrosis, and remodelling [37, 54–56]. All that being associated with the enhancement of pro-fibrotic and pro-osteogenic signals on the VIC [57–59] and a high clinical impact [60, 61]. The higher amounts of inflammatory infiltrates in stenotic male-derived AVs [18] may also contribute to the higher yields of NGAL found in men. Nevertheless, the VIC is per se a sex-specific source of secreted NGAL.

NGAL has gained interest in the last years for its ability to accelerate ECM breakdown while interacting with MMP-9. Recently, HIF-1α up-regulation in hypoxia-challenged VICs was associated with NGAL and MMP-9 expression, and TGFβ signalling [21]. Genetic deletion of *Lcn2* or injections of a NGAL neutralizing antibody protected against abdominal aortic aneurysm, with lower neutrophil infiltration and diminished MMP activity [23]. In our calcifying male-derived VICs, MMP-9 expression and activity were up-regulated, whilst TIMP-1 and -2 were strongly down-regulated (data not shown). Only the latter was further enhanced by rhNGAL and was overall prevented by the pharmacological blockade of NGAL, only in calcifying VICs. Interestingly, the intracellular depletion of NGAL was significantly associated with a high dysbalance of the ECM remodelling markers (high MMPs and low TIMPs). Uncoupling of bone resorption is typical in heterotopic bone formation [62, 63]. Our results may suggest that intracellular NGAL is necessary for the homeostasis of the VIC and that changes in endogenous NGAL modulate the cellular responses to stress in specific tissue and pathologic manners.

The ability of NGAL to induce fibroblast and osteoblast differentiation has been suggested in both hematopoietic and bone marrow cells during myelofibrosis [64]. Indeed, rhNGAL up-regulated osteopontin in calcifying male-derived VICs, and that was abolished by the pharmacological inhibition of NGAL with GP-1. Silencing of NGAL, to mimic the early and specific intracellular depletion of NGAL reported in calcifying male-derived VICs, was found to increase the expression of osteopontin, *RUNX2* and *BMP2* mRNAs. We speculate that intracellular NGAL depletion seen in calcifying male-derived VICs might be both a feature of the metabolic shift fundamental for osteoblast differentiation [65–67] and a maladaptive response to pro-calcific microenvironments. The latter is supported by the effects of intracellular NGAL and its pharmacological inhibition on VIC’s inflammation, oxidative stress, and impaired ECM remodelling. Such paradoxical effects of NGAL were previously reported in kidney disease. Pharmacological blockade of NGAL has proven to be notably beneficial to ameliorate and prevent the fibrotic and inflammatory responses in humanized in vitro and in vivo models of heart and kidney disease [26]. However, in acute kidney disease inhibiting NGAL up-regulation carries severe detrimental effects [26].

## Limitations

There are limitations in our study. The hormone status of the patients was not evaluated in our study. Although all women recruited were post-menopausal, we cannot rule out additional effects of the hormone status on the expression of NGAL and vice versa during premenopausal stages [47, 68, 69]. To provide evidence on the association of NGAL and the sex-related pathogenesis of calcific AS our studies were conducted on AVs from patients with AS. Non-calcific AVs were not used as controls. Nevertheless, the studies performed on AVs sectioned into fibrotic and calcific areas significantly showed an association with NGAL and the degree of calcification, being further validated by regression analysis of NGAL content and calcium score in AVs. Moreover, our in vitro studies were conducted after 4 days of high phosphate-challenge in male-derived VICs evidencing an early up-regulation of inflammation, oxidative stress, ECM remodelling and osteogenic cues. Additional studies on long-term VIC calcification would be appropriate to parallel the advanced calcification stages of our clinical cohort studies.

## Conclusions

NGAL is over-expressed in AVs from men as compared to women. NGAL expression in the AVs of AS patients is associated with inflammation, oxidative stress, osteogenesis, and calcification all of which are relevant to the calcific phenotype of men with AS. Furthermore, VICs are a source of NGAL in AS and in vitro exhibit similar sex-dependent disparities as these reported in explanted AVs. Modulation of NGAL in calcifying male VICs supported different pathological cues depending on the subcellular location. Pharmacological blockade of NGAL with GP-1 (GPZ614741), but not NGAL silencing, ameliorates and prevents the inflammation and remodelling of the calcifying male VIC. The challenge now is to shed light onto the potential of NGAL as an actual druggable target and as marker of AS phenotypes.

## Perspectives and significance

Our study reveals that NGAL exerts differential effects in men and women in AS and permit to speculate on the future research directions. As seen in other forms of cardiovascular disease, the pharmacological blockade of secreted NGAL emerges a promising therapeutic solution to alleviate the expression of inflammatory and matrix remodelling markers in men.

Despite the growing number of publications studying the sex-related differences underlying the pathogenesis of AS, female sex remains being overlooked. The progressive up-regulation of intracellular NGAL in HP-challenged female-VICs, less calcific than male’s, is certainly intriguing and its consequences remain an open question. Further studies will be required to fully understand if the up-regulation of intracellular NGAL in female VICs is associated with a weakened and delayed response to pro-calcific challenges.

## Supplementary Information

**Additional file 1: Table S1**. List of primary antibodies and working concentrations.** Table S2**. List of primers.

**Additional file 2: Figure S1. **Effect of HP in VICs isolated from men and women donors. Scatter dot plot of calcium deposits in primary cultured VICs challenged with HP (2.6mM Pi) (A). Representative immunoblots for intracellular NGAL expression in male and female-derived VICs (D). **** p < 0.0001. Ctrl, control; NGAL, Neutrophil Gelatinase-Associated Lipocalin. **Figure S2. **Effect of NGAL silencing on control VICs from men donors undergoing elective surgical AV replacement. NGAL silencing was evidenced at transcript and protein levels in control conditions (A & B). Scatter dot plots for the relative expression of NGAL codifying gene (*LCN2*) in control male-derived VICs (A). Scramble control group was used as calibrator for the fold-change calculations. GAPDH, 18S, HPRT and ACTA2 were used as housekeeping genes. Representative immunoblots for NGAL expression in man-derived VICs (B). Protein lysates were harvested at day 4 and were assayed into native SDS-PAGE. Protein quantifications were normalized to Stain free. Markers of inflammation (C), ECM remodelling (D) and osteogenesis (E) were assessed by ELISA in cell supernatants from siNGAL VICs under control conditions. Osteogenic markers *RUNX2* and *BMP2* were analysed at the mRNA transcript level as well as the quiescent VIC marker *LECT1* (chondromodulin-I) (F). AU, arbitrary units; *, p < 0.05; **, p < 0.01; ***, p < 0.001; p < 0.0001. *LCN2*, NGAL codifying gene; CCL2, chemokine (C-C motif) ligand 2 (CCL2) or monocyte chemoattractant protein 1 (MCP1); MPO, myeloperoxidase; Gal-3, galectin-3; MMP, metalloproteinase; TIMP, tissue inhibitor of metalloproteinases; BMP, bone morphogenetic protein; *LECT1*, chondromodulin (Chm)-I codifying gene;, periostin; *RUNX2*, Runt-related transcription factor 2. **Figure S3**. Effect of exogenous NGAL regulation in control VICs isolated from men donors. Markers of inflammation (A), ECM remodelling (B) and osteogenesis (C) were assessed by ELISA in cell supernatants from male-derived VICs silenced for NGAL and under control conditions. All comparisons have been represented as a fold change relative to Control condition. **, p < 0.01;. CCL2, chemokine (C-C motif) ligand 2 (CCL2) or monocyte chemoattractant protein 1 (MCP1); MPO, myeloperoxidase; Gal-3, galectin-3; TNF-a, tumour necrosis factor-a; IL, interleukin; MMP, metalloproteinase; TIMP, tissue inhibitor of metalloproteinases; BMP, bone morphogenetic protein.

## Data Availability

From request to the corresponding author.
