# Supplementary material for: Sex-dependent expression of neutrophil gelatinase-associated lipocalin in aortic stenosis
Source: Biol Sex Differ. 2022 Dec 12;13:71. doi: 10.1186/s13293-022-00480-w (PMC9743642; doi:10.1186/s13293-022-00480-w)
Supplement: Supplementary file 1 — Additional file 1: Table S1. List of primary antibodies and working concentrations. Table S2. List of primers. [file 13293_2022_480_MOESM1_ESM.docx]

**Additional file 1**

**Table S1. List of primary antibodies and working concentrations**

WB, western blotting; IHC, immunohistochemistry

**Table S2. List of primers**
