# Supplementary figures and images for: Sex-dependent expression of neutrophil gelatinase-associated lipocalin in aortic stenosis
Source: Biol Sex Differ. 2022 Dec 12;13:71. doi: 10.1186/s13293-022-00480-w (PMC9743642; doi:10.1186/s13293-022-00480-w)

**A**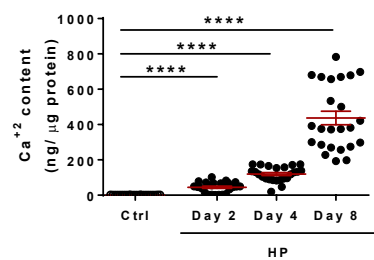**B**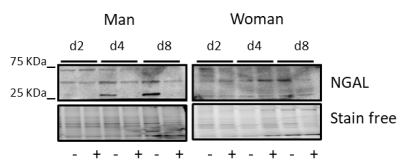

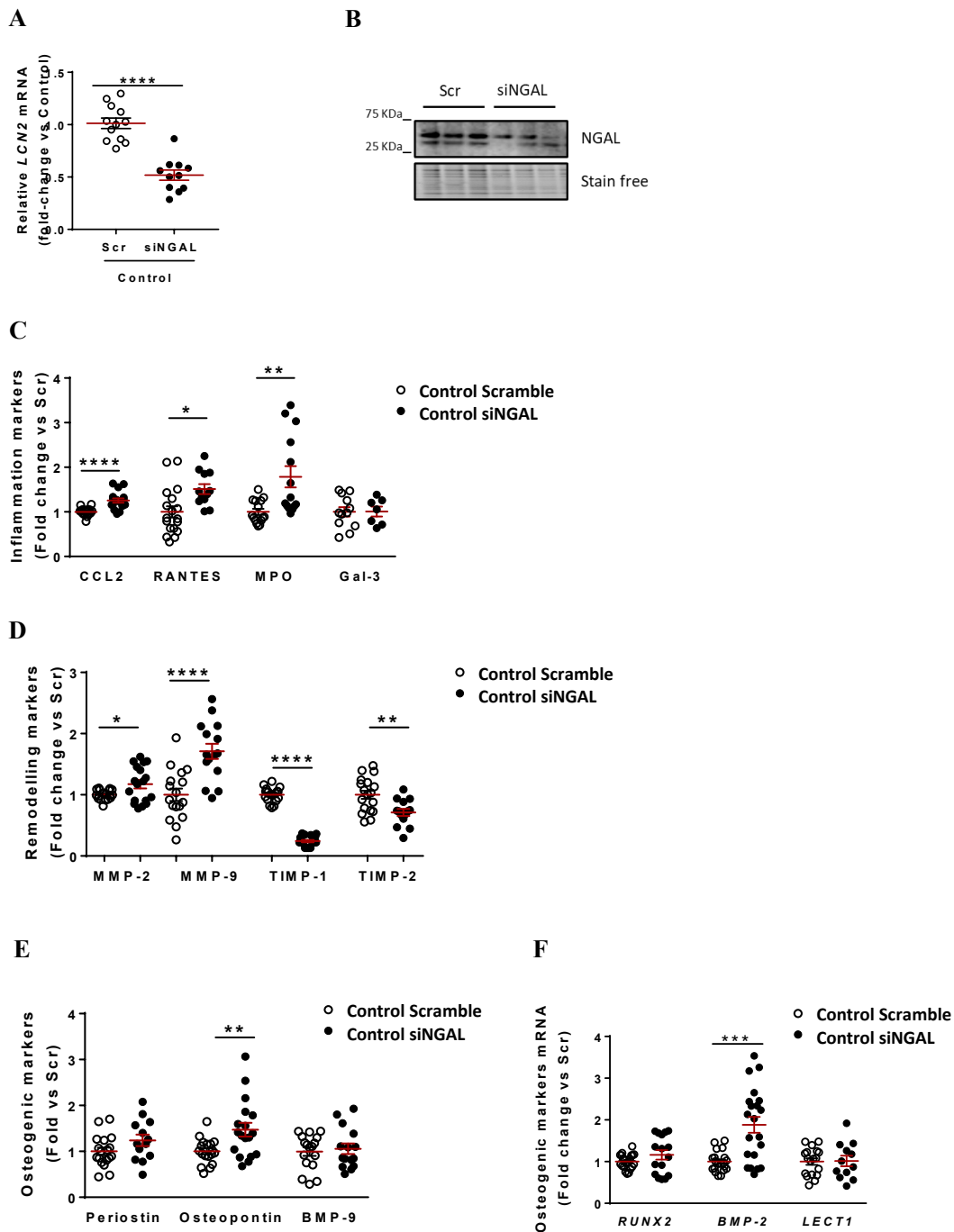

A

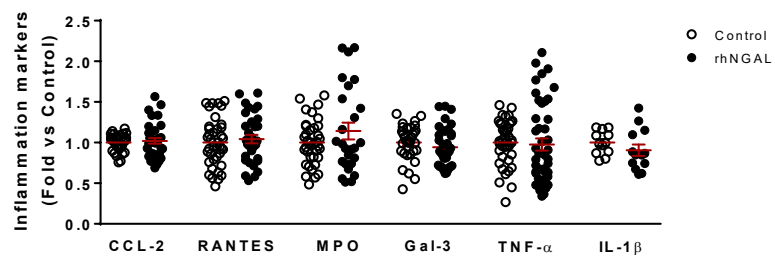

B

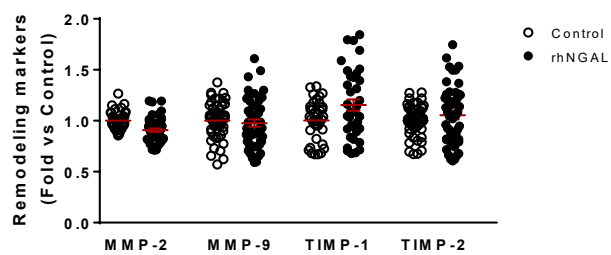

C

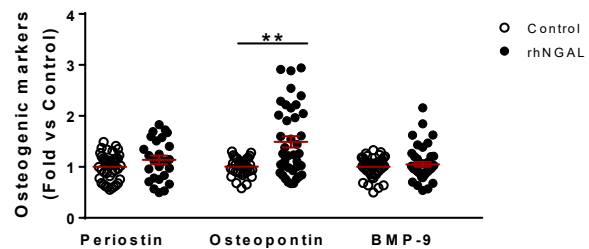

Supplement: Supplementary file 2 — Additional file 2: Figure S1. Effect of HP in VICs isolated from men and women donors. Scatter dot plot of calcium deposits in primary cultured VICs challenged with HP (2.6mM Pi) (A). Representative immunoblots for intracellular NGAL expression in male and female-derived VICs (D). **** p < 0.0001. Ctrl, control; NGAL, Neutrophil Gelatinase-Associated Lipocalin. Figure S2. Effect of NGAL silencing on control VICs from men donors undergoing elective surgical AV replacement. NGAL silencing was evidenced at transcript and protein levels in control conditions (A & B). Scatter dot plots for the relative expression of NGAL codifying gene (LCN2) in control male-derived VICs (A). Scramble control group was used as calibrator for the fold-change calculations. GAPDH, 18S, HPRT and ACTA2 were used as housekeeping genes. Representative immunoblots for NGAL expression in man-derived VICs (B). Protein lysates were harvested at day 4 and were assayed into native SDS-PAGE. Protein quantifications were normalized to Stain free. Markers of inflammation (C), ECM remodelling (D) and osteogenesis (E) were assessed by ELISA in cell supernatants from siNGAL VICs under control conditions. Osteogenic markers RUNX2 and BMP2 were analysed at the mRNA transcript level as well as the quiescent VIC marker LECT1 (chondromodulin-I) (F). AU, arbitrary units; *, p < 0.05; **, p < 0.01; ***, p < 0.001; p < 0.0001. LCN2, NGAL codifying gene; CCL2, chemokine (C-C motif) ligand 2 (CCL2) or monocyte chemoattractant protein 1 (MCP1); MPO, myeloperoxidase; Gal-3, galectin-3; MMP, metalloproteinase; TIMP, tissue inhibitor of metalloproteinases; BMP, bone morphogenetic protein; LECT1, chondromodulin (Chm)-I codifying gene;, periostin; RUNX2, Runt-related transcription factor 2. Figure S3. Effect of exogenous NGAL regulation in control VICs isolated from men donors. Markers of inflammation (A), ECM remodelling (B) and osteogenesis (C) were assessed by ELISA in cell supernatants from male-derived VICs silenced [file 13293_2022_480_MOESM2_ESM.pdf]
